# Disentangling change across the time and true stability of employees’ resilience using latent state model

**DOI:** 10.1186/s12888-022-04294-3

**Published:** 2022-10-20

**Authors:** Lucie Ollis, Mark Cropley, David Plans, Hugo Cogo-Moreira

**Affiliations:** 1 School of Health Sciences, University of Surrey, Guilford, Surrey UK; 2 School of Psychological Sciences, University of Surrey, Guilford, Surrey UK; 3 Department of Psychology, Royal Holloway, University of London, Egham, Surrey UK; 4 Department of Education, ICT and Learning, Østfold University College, Halden, Norway

**Keywords:** Resilience, Trait, State, Latent-state model, Cohort study, Stability

## Abstract

There is debate within the literature about whether resilience should be considered a stable character trait or a dynamic, changeable process (state). Two widely used measures to assess resilience are the Connor-Davidson Resilience Scale (CD-RISC) and the Resilience Scale for Adults (RSA). The aim of this study was to evaluate the true stability (invariance) and change across time in resilience captured by these two measures. Using the perspective of Latent State-Trait theory, the aim was to decipher if the CD-RISC and the RSA are more trait-like or more state-like and to address whether true differences in resilience between participants increased (or decreased) across time. In this longitudinal study, UK-based employees (*N* = 378) completed the CD-RISC (10-item version) and the RSA (33-item version, aggregated and analyzed under six parcels) at three occasions over six months. A latent-state model and latent-state model with indicator specific residual factors were utilized. The analysis suggested that both questionnaires capture trait and state components of resilience. These results contribute to the discussion about how resilience scales are measuring change and stability, and how we define resilience as a more trait-like or state-like phenomena. The findings also highlight the issue of what resilience scales are measuring and whether resilience is a quantifiable construct.

**Supplementary Information:**

The online version contains supplementary material available at 10.1186/s12888-022-04294-3.

A recent concept analysis [1] defined individual resilience as a dynamic concept which describes someone’s ability to successfully adapt and function despite psychological, sociological, cultural and/or physical adversity. Resilience has received particular attention in recent years due to ambiguities in terminology and definitions, inconsistencies in experiences of resilience of those at risk, instability of the concept and a lack of attention to theoretical concerns [2, 3]. There has been much discussion and disagreement within the literature about whether resilience should be considered a character trait which remains relatively stable over time or a dynamic process (state) which varies across time [4, 5].

In terms of Latent State-Trait Theory, a trait is described as the characteristics of a person, whereas psychological states of a person may vary across time depending on exposure to changing situations [6]. Some researchers support the notion that resilience is a relatively stable character trait [6–9], while most researchers argue that resilience is a developmental state which is not static, fixed, or immutable [3, 10–14]. Resilience is thought to be variable and situation-specific, operating at multiple levels within a person and influenced by interactions between our physical, social, and environmental resources. Windle [14] believes individuals’ assets, resources and environment facilitate resilience and the ability to ‘bounce back’ after experiencing adversity. For example, an individual may adapt well to workplace stress but may not fare so well with problems in their personal life or in relationships [15].

Studies investigating changes in resilience have found evidence for resilience as a trait. For example, Crane and Searle [16] suggested resilience remains relatively stable over time as there was a strong positive correlation in employees' resilience between the two occasions in time. In addition, change scores were used to infer differences in resilience over time and found no significant differences in resilience between the two occasions in a sample of hospital employees [17]. Although in both studies the findings supported the notion of resilience as a trait, the ‘change’ in resilience was inferred using correlations or change scores and only included two occasions of data collection. On the other hand, there is limited empirical research to support the claim that resilience is a dynamic process which changes over time (e.g. [3, 10, 12–14]). Discussions have been theoretical in their nature or researchers have only assessed employees’ resilience at two occasions. This could limit the amount of potential variability seen.

The way in which we define resilience underlies how we might measure resilience and therefore any attempt to quantify resilience is intricately linked with issues with the definition presented in the literature [14]. In their review of resilience measures, Smith-Osborne and Whitehill Bolton [18] suggest the Connor-Davidson Resilience Scale (CD-RISC, [19]) and the Resilience Scale for Adults (RSA, [20]) are the two most reliable instruments for use in longitudinal studies with adult populations.

The theoretical basis of the CD-RISC is based upon the coping, adaptation, and stress literature [11, 21, 22] and is a measure of an individual's ability to cope with stress [19]. It has been suggested the original version of the CD-RISC had an unstable factor structure [23] and Campbells-Sills and Stein made a series of empirically driven modifications to create a revised version, the CD-RISC 10. The 10-item version is unidimensional and assesses similar components of resilience to the original scale e.g., an individuals’ ability to tolerate change, personal problems, illness, pressure, failure, and painful feelings [23].

The RSA was designed to assess protective factors present in individuals that were deemed to be important for the recovery and maintenance of good mental health [20]. The authors modified the original 45-item version of the RSA [20] and created a 33-item scale that included six factors: personal strength/perception of self, personal strength/perception of future, structured style, social competence, family cohesion, and social resources [24]. General resilient factors are assumed by the authors to be relatively stable over time [25]; however, in terms of psychometrics and Latent State-Trait Theory (described briefly below), there is a lack of evidence for such relative stability. The definition of resilience given by Friborg et al. [20, 24] views resilience as a multidimensional construct in which people possess the capacity to combat adversity or misery.

For the authors of the CD-RISC and the RSA [19, 23], resilience is thought to be modifiable (a state). As with the CD-RISC, the authors of the RSA do not explicitly state whether their scale is measuring trait or state aspects of resilience, but the wording of the items evaluate both long term and momentaneous behavior. For example, researchers may ask about how participants feel/think in the present moment (assessing current mood states) or how they feel/think more generally (assessing traits) [26]. The way in which statements are presented in psychometric scales will have an impact on whether we are assessing a concept as a state or trait. The CD-RISC 10 asks individuals to answer statements based on how they apply to them over the last month. All ten statements ask how individuals cope with changes, adversity, and the pressures of life, in general. Although the statements are generic and one would assume that this would measure more trait-like components of resilience are being assessed, individuals are asked to think about the *last month* when selecting their answers, suggesting there could be changes over time. On the other hand, the RSA does not include a timescale in the instructions of the scale and the statements are based around assessing the protective factors which may foster resilience rather than the ability to cope (as with the CD-RISC). The statements of the RSA are based more around the external circumstances which may affect one’s ability to withstand adversity, for example family cohesion and social resources. Although these factors may well change over time, the wording of the questionnaire is nevertheless generic.

The authors of the CD-RISC and RSA describe resilience as a multidimensional construct influenced by a variety of factors, but there is a lack of evidence regarding how both commonly used scales track resilience across time. In the area of psychometrics, terms such as traits, states, and changes might be statistically formalized via the Latent State-Trait (LST) theory, which is “…a longitudinal conceptualized measurement theory that allows us to separate true individual differences from individual differences due to random measurement error” [27] (p. 3). However, under the classical LST theory (see [6, 28]), the participant is considered as a static entity [27] (p. 4) not interacting over time. In the context of behaviors and latent constructs such as resilience, LST theory defines state, trait, and state residual latent constructs in terms of probability theory, allowing researchers to decompose such sources of information under longitudinal designs.

LST theory was revised by Steyer et al., [29] who introduced the notion of a *person-at-time*, where subjects interact with the environment and consequently, they are not the same across time (for more details see [29]). As described by Steyer et al., [29], the latent state-trait theory considers that “…observations are fallible, they never happen in a situational vacuum, they are always made using a specific method of observations, and there is no person without a past” (p. 71). This means that people constantly experience things differently so that psychologically, the person at a specific time point is not necessarily the same as the person at the subsequent time point. In terms of the theory of resilience, there is debate about whether what is being measured should be either a state or trait. Statistically, such a dichotomy is not an issue as noted by Hertzog and Nesselroade [30] where they state that “psychological variables may contain both state-like and trait-like components” (p. 105). The original LST theory first presented by Steyer et al. [31] considered traits as rigid which is quite unrealistic in practice because the concept of a trait variable is dependent only on person-person characteristics, where the person per se is not expected to go through changes across time given faced experiences or situational events.

In line with Nesselroade [30], state variability might be described as a process that involves reversible short-term changes in individuals’ true scores (e.g., ups and downs which are time- or situation-specific in individuals) around an invariant set-point, the trait value. Also, traits might change across time, but they are characterized by a more enduring component. LST theory provides quantitative evidence regarding the behavior of the assessment within a given time-lag, giving the researcher insights about what they are capturing, considering their longitudinal design. Moreover, the recent LST-R theory considers the fact that individuals constantly change across time, interact with time specific situations, and previous experienced situations also impact current behavior; therefore, states and traits are dynamic [29].

According to Karaırmak and Figley [32] no study has attempted to test resilience as a dynamic state or a relatively stable trait over time. Of those studies that have investigated changes in resilience over time, many researchers have not used appropriate models to separate random measurement error from true score variance, which allow us to evaluate true stability and changes across time. Therefore, the main aim of this research is focused on measurement-related issues for homogeneity of indicators of resilience and the degree of measurement invariance. In other words, how the measurement model underlying the indicators of both scales is invariant across time. By measuring invariance, we are invoking the concepts of invariance typically applied in multigroup confirmatory factor analysis, but here, given the longitudinal design, such a concept is applied across time to understand whether the psychometric features of both scales remain stable across three measurement occasions. From the results of measurement invariance, change, stability, and derivations, one might determine if the scales are tracking more trait-like or state-like phenomena. The 10-item version of the CD-RISC [23]and the 33-item version of the RSA were used [24].

For the RSA, the analyses were conducted at parcel level. We used a latent-state model (LSM) and latent state model with indicator specific residual factors (LS-IS), following the model specifications suggested by Eid et al. [33], adding longitudinal invariance constraints to the RSA *parcel* parameters (intercepts and factor loadings) and for the CD-RISC at item level parameters (thresholds and factor loadings) to evaluate invariance across time. The RSA parcels are the six subscales (composite scores) derived from the sum of the item's scores.

The latent state models, once found to fit well, might guide researchers to more complex longitudinal modelling, such as adding autoregressive effects and models where traits are formally specified (for an example of latent-state-trait with autoregressive effect see [34]). In the present longitudinal study, the CD-RISC and RSA were administered three times over the course of six months. Two questions were addressed:

1. Are both scale measurements invariant across time?
  One practical implication of lack of invariance across time might be that scores across time might not be directly comparable depending on the level of measurement invariance achieved. Therefore, researchers would not be advised to test simple mean differences in the scores across time.
2. How does resilience change over time as measured by these two scales?
  This question brings elements for understanding if true scores of the participants are increasing across time (or not), if true differences between the subjects are increasing (or not) across time, and if the scales and their underlying constructs are tracking more trait-like or state-like constructs.

Determining whether a construct measured by a scale is a state or a trait, or both, is a fundamental question and only can be addressed under longitudinal designs. Because latent-state models provide insights regarding what and how we are measuring a phenomenon across time, one might disentangle if the effect across time is an effect on the predisposition (traits) or just attenuation in ups-and-downs (occasion-specific events). In the context of randomised trials, for example, this might be fundamental to see if the random allocation status has effect on traits (enduring feature) or states (occasions-specific events). Moreover, LSMs depart from time invariance testing and scales without such a longitudinal invariance feature pose threat to a simple ordinary pre- and post-mean scores comparison via ordinary tests as t-test, repeated ANOVA and so on. At least strong invariance level is required (see Method section) to guarantee that the meaning of the construct under evaluation is longitudinally preserved, and therefore, changes and dynamics across time might be evaluated.

Beyond the t-tests and repeated ANOVAs, when working under LSMs, mean differences between the continuous latent states allow us to understand if the true score of resilience is changing across time; this is conducted by imposing constraints at mean-level of the state variables. In addition, the stronger the correlations are between the state variables, the more trait-like the constructs are, whereas smaller correlations indicate that the constructs are more state-like.

## Method

### Sample

Informed consent was obtained from all participants. They were employees 18 years old and above, English speaking and working at least 30 h per week in a role based in the UK. It was outlined in the participant information sheet that individuals should not participate in the study if they had any stress-related health conditions or if they thought answering questions about their health and well-being could have a negative impact on them. Participants were recruited on a voluntary basis through social media platforms (e.g., Twitter, LinkedIn), emails to the researcher’s contacts, posters displayed at a university (not just aimed at university staff) and through snowball sampling. Participants were offered the chance to be entered into a prize draw for one of two £25 vouchers for completing all three stages of this longitudinal study. Data was collected between April 2018 and May 2019.

### Participants

In total, 415 participants consented to complete the baseline measures (Occasion 1), 180 took part in the 3-month follow-up (Occasion 2) and 136 individuals responded at the 6-month follow-up (Occasion 3, 76% retention). Thirty-seven participants were removed from Occasion 1 as the questionnaire was incomplete or the participants were not eligible for the study. Two further participants were removed from Occasion 2 as the questionnaire was incomplete. Finally, two participants were removed from Occasion 3 as we were unable to match the data from the email address provided or the questionnaire was incomplete. Included in the final analysis were 378 participants for Occasion 1, 178 for Occasion 2 (47% retention) and 134 for Occasion 3 (75% retention).

The average age of participants at Occasion 1 was 34.05 years (*SD* = 12.87) including 247 women (65.3%) and 130 men (34.4%). The participants identified as White British (*N* = 272, 72.2%), White ‘Other’ (*N* = 54, 14.3%), White and Asian (*N* = 9, 2.4%), Indian (*N* = 7, 1.9%), Black or Black British African (*N* = 6, 1.6%) or Chinese (*N* = 6, 1.6%). For level of education, 158 participants (41.8%) had a postgraduate degree, 160 participants (42.3%) had an undergraduate degree and 59 participants (15.6%) had GCSE’s or A-Level equivalents. Participants were employed in several different sectors including (but not limited to): Teaching or education (*N* = 55, 14.6%), business, consulting, or management (*N* = 53, 14%), research or science (*N* = 51, 13.5%), healthcare (*N* = 32, 8.5%) and accounting, business, or finance (*N* = 19, 5%).

### Instrument

Participants’ individual resilience was assessed using the 10-item version of the CD-RISC [23] and the 33-item RSA [24]. These are outlined in the introduction in more detail. The CD-RISC is based on one factor of resilience and uses a 5-point Likert scale, scored from 0 (not true at all) to 4 (true nearly all of the time). For copyright reasons, example items of this scale cannot be reproduced. The main features of this scale reflect an individuals’ ability to tolerate change, personal problems, illness, pressure, failure, and painful feelings.

The RSA includes six subscales representing six different elements of resilience, utilizing a semantic differential scale (five points). Examples include personal strength/perception of the self (six items e.g., My abilities: I strongly believe in, I am uncertain about), personal strength/planned future (four items e.g., My goals for the future are: unclear, well thought through), structured style (four items e.g., I am good at: organizing my time, wasting my time), social competence (six items e.g., I enjoy being: together with other people, by myself), family cohesion (six items e.g., My family is characterized by: disconnection, healthy coherence) and social resources (seven items e.g., The bonds among my friends is: weak, strong). For the analysis, the six subscales of the RSA were analyzed as six separate parcels.

### Design

Favourable ethical opinion was granted by the University of Surrey Ethics Committee for this project (Reference: UEC 2018 017 FHMS). The study was pre-registered on the Open Science Framework (osf.io/ezgrf), stipulating the measurement of multiple other factors in addition to assessment of resilience via the CD-RISC and the RSA. However, the purpose of this paper was to focus on whether the scale measurements are invariant across time and whether resilience scales can track change and stability (according to changes in self-reported resilience). Therefore, the remaining data was not included and there are no current plans to publish or analyze the remaining data.

A within-subjects, repeated measures design was used to address the research questions. Once the participants had accessed the research study on Qualtrics (www.Qualtrics.com) through the advertisements, they were asked to read a participant information sheet and give informed consent. Participants then completed demographic information including details about their: gender, age, ethnicity, occupation, working pattern, how long they had been in their current role and education level. The demographic query was followed by completion of the CD-RISC and the RSA. Thereafter, participants were debriefed and asked to provide an email address for the follow-up questionnaires. This process was repeated for Occasion 2 and 3. Each stage took participants approximately 10 min to complete and participants were actively participating in the study for approximately 6-months.

### Statistical analysis

For a better understanding of the stability and change in resilience, we used the LSM and a less restricted form of the LSM called the latent-state model with indicator specific residual factors (LS-IS). The models were used to evaluate if the two resilience scales (the CD-RISC and the RSA) were more trait-like or more state-like, and to evaluate whether true differences in resilience between subjects increased (or decreased) across time. The LSM is a more restrictive model that assumes that the observed measures fit well to a unidimensional model. The LS-IS, introduced by Eid et al. [33], allows more complex longitudinal covariance structure “…in which the same measures are more highly correlated with itself across time that with the other measures of the same LS variable” (p. 136). All the models were run in Mplus version 8.6. Syntaxes for both models with annotation regarding the step-by-step of the invariance testing are available in full for Mplus, in Supplementary materials 1 and 2.

Both models are derived from LST theory, which was revised in 2015 by Steyer et al. [29] as decomposing an observed variable (i.e., items of a scale or a composite score) on a given occasion of measurement into a latent state (characterizing the momentary state) and an error variable. The latent state variable is decomposed into two sources of information (i.e., variances): A latent trait variable (representing individual disposition) and a latent occasion-specific state residual variable (representing a deviation between the person’s dispositions at a given moment, in other words, the extent to which it is the person’s reaction to a given situation given his/her trait). Trait and occasion-specific state conceptions come from four fundamental facts described by Steyer et al. [29] as follows: “…1) Observations are fallible, 2) they never happen in a situational vacuum, 3) they are always made using a specific method of observation, and 4) there is no person without a past” (p. 93).

For the LSM and LS-IS models (when LSM did not fit or returned an improper solution, the LS-IS model was specified), a series of hierarchical restrictions to the item’s parameters (i.e., factor loadings, thresholds/intercepts, residual variances, and other model parameters described below) were imposed to understand the measurement invariance for both scales. We followed the steps recommended by Liu et al. [35] in the case of ordered-categorical items (i.e., at item-level of CD-RISC) and followed the didactic Mplus syntax examples provided by Geiser [36] for continuous indicators (i.e., six parcels of RSA).

The following nested models for ordered-categorical indicators were tested following the adapted version of Liu et al. [35] model’s specification, but without the residual correlations between items across time, because LS-IS does already consider individual item-effects across time. Moreover, for LSM, adding the correlations between items’ residuals across time changes the interpretation of the latent state correlations and decomposition of longitudinal associations in this model is not clear. The nomenclature used by Liu et al. [35] was maintained (from the less restrictive to the most restrictive): a) baseline model where the same general pattern of factor loadings holds across time, b) the loading invariance model by constraining factor loadings identical across time, c) threshold invariance model constraining, for each indicator, the threshold level of going from one response category to the next is identical over time, d) the unique factor invariance model where all unique factor variances are equal over time, e) lagged covariances of the continuous latent responses over time, and lastly, g) taking all the previous and adding the means of the continuous latent variable were restricted to be equal across time. Given sparse data problems (number of thresholds changing across time), the first and second categories of the original five-point Likert scale indicators of CD-RISC were merged.

Under the WLSMV estimator of Mplus, the DIFFTEST function was used to evaluate the difference between nested ordered-categorical confirmatory factor analysis (CFA) models. Under MLR estimator, the chi-square difference testing using the Satorra-Bentler Scaled Chi-Square [37] was used to compare nested models together with Akaike’s Information Criteria (AIC) and Bayesian Information Criteria (BIC). Via means, variances, and correlations of the latent state variables we examined stability and change across time, where:

- Mean difference between latent state variables indicate whether on average, true scores of resilience increased (or decreased) across time [36] (see p. 125);
- Changes in the variance of the latent state variables evaluates whether the true scores between individuals increased (or decreased) across time [36] (see p. 125)
- Correlations between the latent state variables show to what extent the rank order of individuals remained the same or changed across time; the stronger the correlation, the more trait-like constructs [36] (see p. 125).

For the RSA and its six parcels (i.e., personal strength/perception of self, personal strength/perception of future, structured style, social competence, family cohesion, and social resources), we followed the Widaman and Reise [38] nomenclature in terms of hierarchical measurement invariance testing: a) configural invariance, b) weak invariance, c) strong invariance, d) strict invariance, e) strict invariance with equal latent state factors means across time, f) strict invariance with equal latent state factor means and their variances across time, and g) lastly adding constraints (equality) to the latter model at the covariance between the latent state factors.

We used the following model fit indices to evaluate our model based on the recommendations of Schermelleh-Engel et al. [39]: χ^2^-test, comparative fit index (CFI), Tucker-Lewis Index (TLI), root mean square error approximation (RMSEA), and Standardized Root Mean Square Residual (SRMR). The CFI and TLI should be greater than or equal to 0.97. A RMSEA value less than or equal to 0.05 indicates a good approximate model fit. The *p*-value of the corresponding test of approximate fit should be equal to or smaller than 0.05. Finally, a SRMR smaller than 0.08 would indicate a good fit.

In terms of estimators, for the ten Likert scales of the CD-RISC, we used weighted least square with mean and variance adjusted (WLSMV) chi-square, which uses the diagonal of the weight matrix in the estimation and the full weight matrix to compute standard errors and chi-square. This is the default estimator in Mplus for categorical-ordered variables (i.e., Likert scale). WLSMV is a limited-information estimator that deals with missing data using pairwise deletion, which accommodates different sample sizes for different pairs of dependent variables [40]. For the RSA, we used a robust maximum-likelihood estimator, because we were dealing with six parcels, which are considered continuous variables. Therefore, differently from the CD-RISC, the analysis of the RSA scale was not conducted at item-level, but the items’ composite score for the six subscales (parcels). Under maximum-likelihood estimator missing data are dealt with full information maximum likelihood assuming that the missingness mechanism across time is missing at random.

## Results

### LSM for CD-RISC and RSA models fit

**Fig. 1:**
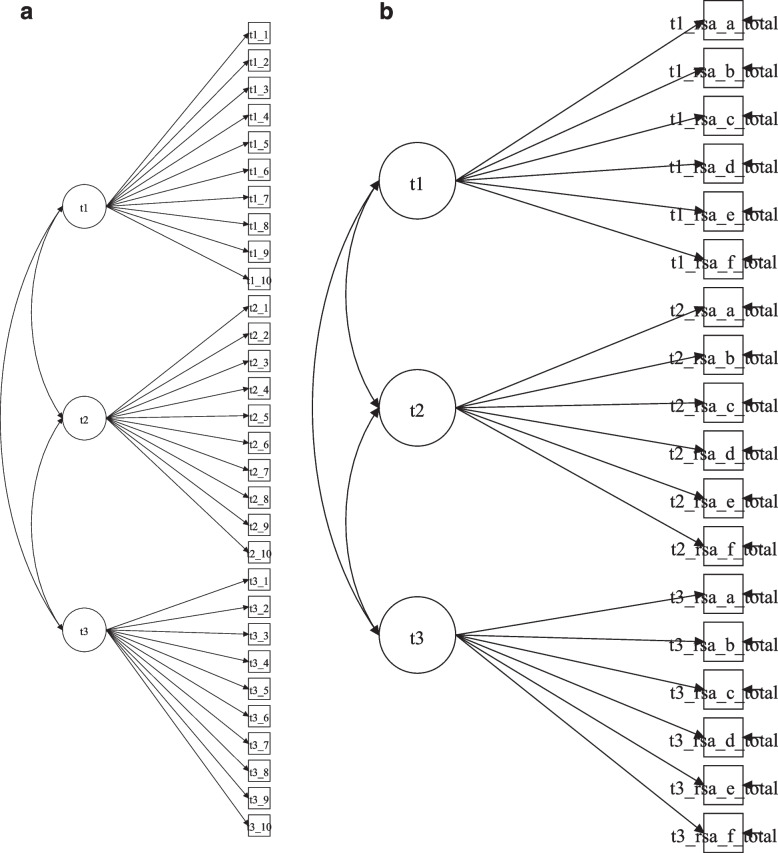
**a **Diagram Representing Latent State Model for CD-RISC. **b **Diagram Representing Latent State Model for RSA. **a **Squares represent ordered-categorical items of CD-RISC and ovals (on bottom, “TAU”) latent continuous state variables and indicator specific latent factors (on top). T1_1 represents the time 1 assessment for the first item of CD-RISC “adaptation to changes”; T1_2 represented the time 1 assessment for the second item of CD-RISC; T2_1 represents the time 2 assessment for the first item. Therefore, the first letter and number are related to the wave of assessment and after the underlying we have the item number. **b **Squares represent parcels of RSA and ovals (on bottom, “TAU”) latent continuous state variables and indicator specific latent factors (on top). T1_1 represents the time 1 assessment for the first parcel (subscale) of RSA; T1_2 represented the time 1 assessment for the second parcel (subscale) of RSA; T2_1 represents the second assessment for the parcel 1 (subscale) of RSA; Therefore, the first letter and number are related to the wave of assessment and after “_”we have the parcel number

Under the LSM approach, the CD-RISC (Fig. 1A) returned a poor model fit (χ^2^_(402)_ = 886.118, *p*-value < 0.001, RMSEA = 0.057 (90% Confidence Interval [CI] = 0.051 to 0.062), Close fit = 0.017, CFI = 0.906, TLI = 0.898). The LSM for the RSA (Fig. 1B) returned an improper solution given the latent variable covariance matrix (psi) is not positively definite.

**Fig. 2:**
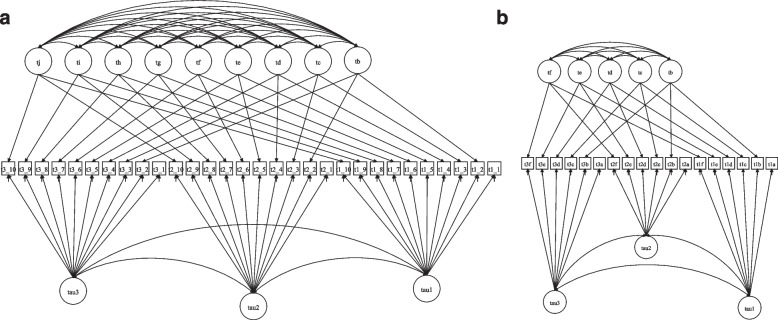
**a **Diagram Representing Latent State with Indicator Specific Model for CD-RISC. **b **Diagram Representing Latent State with Indicator Specific Model for RSA. **a **Squares represent ordered-categorical items of CD-RISC and ovals (on bottom, “TAU”) latent continuous state variables and indicator specific latent factors (on top). T1_1 represents the time 1 assessment for the first item of CD-RISC “adaptation to changes”; T1_2 represented the time 1 assessment for the second item of CD-RISC; T2_1 represents the time 2 assessment for the first item. Therefore, the first letter and number are related to the wave of assessment and after the underlying we have the item number. **b **Squares represent parcels of RSA and ovals (on bottom, “TAU”) latent continuous state variables and indicator specific latent factors (on top). T1_1 represents the time 1 assessment for the first parcel (subscale) of RSA; T1_2 represented the time 1 assessment for the second parcel (subscale) of RSA; T2_1 represents the second assessment for the parcel 1 (subscale) of RSA; Therefore, the first letter and number are related to the wave of assessment and after “_”we have the parcel number

Therefore, for both assessments, we used a less restrictive modelling approach, the LS-IS (also called residual method or IS factors; see Fig. 2a and b, for CD-RISC and RSA, respectively).

Note that the main difference between the Fig. 1a and 1b in relation to Fig. 2a and 2b is the residual factor was added in which captures a “Method” effect. Items (and parcels) with the same wording or content are loaded onto the same factor.

**Table 1:** The different levels of invariance achieved by the CD-RISC and RSA

						RMSEA							DIFFTEST/Χ^2^δ		
Scale	Modelling approach	Model	Invariance Model	χ^2^(df), *p*-value	Estimate	90% Percent CI	Close fit	CFI	TLI	SRMR	Chi-square value And Model comparison	dfΔ	*p*-value (χ^2^Δ)	AIC	BIC
CD-RISC	Latent State		Baseline	886.118(402), < 0.001	0.057	0.051 to 0.062	0.017	0.906	0.898	0.083					
CD-RISC	LS-IS	1	Baseline	361.012(339), 0.1967	0.013	0.000 to 0.024	1	0.996	0.995	0.045					
	LS-IS	2	Loading Invariance	374.162(357), 0.2556	0.011	0.000 to 0.022	1	0.997	0.996	0.046	13.933 (2 vs 1)	18	0.7334		
	LS-IS	3	Threshold Invariance	414.134(395), 0.2439	0.011	0.000 to 0.022	1	0.996	0.996	0.047	42.044 (3 vs 2)	38	0.3000		
	LS-IS	4	Unique Factor	432.087(415), 0.2715	0.010	0.000 to 0.021	1	0.997	0.997	0.050	19.952 (4 vs 3)	20	0.4609		
	LS-IS	5	Model 4 with equal correlation between the state factors	435.057(417), 0.2613	0.011	0.000 to 0.021	1	0.996	0.996	0.052	2.553 (5 vs 4)	2	0.2739		
	LS-IS	6	Model 5 with t2 and t3 means equal	435.099(418), 0.2721	0.010	0.000 to 0.021	1	0.997	0.997	0.052	0.635 (6 vs 5)	1	0.4257		
	LS-IS	7	Model 6 with equal variance of state factor	464.755(420), 0.0648	0.017	0.000 to 0.025	1	0.991	0.991	0.052	23.151 (7 vs 6)	2	< 0.0001		
RSA	LS-SI	1	Configural invariance	148.617 (117) 0.0257	0.027	0.010 to 0.039	1	0.984	0.979	0.067				21,147.032	21,430.153
	LS-SI	2	Weak invariance	150.508 (127) 0.0758	0.022	0.000 to 0.035	1	0.988	0.986	0.065	25,364 (2 vs 1)	10	0.9903	21,130.634	21,374.433
	LS-SI	3	Strong invariance	163.871 (137) 0.585	0.023	0.000 to 0.035	1	0.986	0.985	0.069	12.8525 (3 vs 2)	10	0.2320	21,123.882	21,328.359
	LS-SI	4	Strict invariance	171.854 (149) 0.0968	0.020	0.000 to 0.033	1	0.988	0.988	0.067	9.2663 4 vs 3)	12	0.6800	21,110.293	21,267.583
	LS-SI	5	Strick invariance, equal LS factor means	178.924 (151) 0.0600	0.022	0.00 to 0.034	1	0.986	0.986	0.070	4.7056 (5 vs 4)	2	0.0951	21,113.755	21,263.18
	LS-SI	6	Strick invariance, equal LS factor means and variances	184.923 (153) 0.0401	0.024	0.005 to 0.035	1	0.984	0.984	0.097	4.0175 (6 vs 5)	2	0.1342	2115.954	21,257.515
	LS-SI	7	Strick invariance, equal LS factor means, variances, and covariances	185.748 (155) 0.0465	0.023	0.003 to 0.034	1	0.984	0.984	0.097	6.2915 (7 vs 6)	2	0.0430	21,114.561	21,248.258

*Note*. *LS-SI* Latent State with indicator-specific factor, *df* Degrees of freedom, *CI* Confidence interval, *CFI* Comparative Fit index, *TLI* Tucker-Lewis index, *SRMR* Standardized root mean square residual, *AIC* Akaike's information criterion, *BIC* Bayesian information criterion

**Table 2:** CD-RISC correlation matrix between its latent state factors and IS factors item 1 (on top) and item 4 (on bottom) as reference

	T1	T2	T3	Item 2	item 3	Item 4	Item 5	Item 6	Item 7	Item 8	Item 9	Item 10
T1	1.000											
T2	0.791	1.000										
T3	0.696	0.671	1.000									
Item 2	0.000	0.000	0.000	1.000								
Item 3	0.000	0.000	0.000	0.362	1.000							
Item 4	0.000	0.000	0.000	-0.199	0.128	1.000						
Item 5	0.000	0.000	0.000	0.069	0.153	0.370	1.000					
Item 6	0.000	0.000	0.000	0.194	-0.026	0.287	0.477	1.000				
Item 7	0.000	0.000	0.000	-0.083	0.067	0.349	0.011	0.281	1.000			
Item 8	0.000	0.000	0.000	0.362	0.177	0.240	0.319	0.535	0.575	1.000		
Item 9	0.000	0.000	0.000	0.510	0.157	0.324	0.370	0.289	0.226	0.586	1.000	
Item 10	0.000	0.000	0.000	0.379	0.203	0.307	0.302	0.326	0.400	0.394	0.849	1.000
	T1	T2	T3	Item 1	Item 2	Item 3	Item 5	Item 6	Item 7	Item 8	Item 9	Item 10
T1	1.000											
T2	0.779	1.000										
T3	0.672	0.602	1.000									
Item 1	0.000	0.000	0.000	1.000								
Item 2	0.000	0.000	0.000	0.958	1.000							
Item 3	0.000	0.000	0.000	0.428	0.483	1.000						
Item 5	0.000	0.000	0.000	0.481	0.449	0.283	1.000					
Item 6	0.000	0.000	0.000	0.407	0.435	0.103	0.523	1.000				
Item 7	0.000	0.000	0.000	0.393	0.287	0.174	0.086	0.315	1.000			
Item 8	0.000	0.000	0.000	0.478	0.521	0.301	0.405	0.581	0.609	1.000		
Item 9	0.000	0.000	0.000	0.616	0.737	0.324	0.467	0.379	0.304	0.640	1.000	
Item 10	0.000	0.000	0.000	0.479	0.607	0.339	0.385	0.389	0.447	0.476	0.864	1.000

Legend: item 1 = I am able to adapt to change; Item 2 = I can deal with whatever comes; item 3 = I try to see the humorous side of problems; item 4 = Coping with stress can strengthen me; Item 5 = I tend to bounce back after illness or hardship; Item 6 = I can achieve goals despite obstacles; Item 7 = I can stay focused under pressure; Item 8 = I am not easily discouraged by failure; item 9 = I think of myself as a strong person; Item 10 = I can handle unpleasant feelings

Table 1 describes the different levels of invariance achieved by CD-RISC and RSA under LS-IS modelling.

### CD-RISC stability and change across time

The CD-RISC achieved a unique invariance level because model 6 versus model 5 in Table 1 had a chi-square difference test which was not statistically significant (Diff test χ^2^ (1) = 0.635, *p*-value = 0.4257). Therefore, we conclude that the expected means, variances, and within-wave covariances of the continuous latent responses are entirely attributable to changes in the latent common factors over time (see [35], page 12). indicating that we have a lack of evidence on the mean difference between state latent factors across time. The standardized mean differences between the state latent factor t2 and t1 (delta = 0.122, SE = 0.076, *p* = 0.109) and t3 and t1 (delta = 0.107, SE = 0.066, *p* = 0.106) were not statistically significant, nor t2 against t3. Consequently, this indicates the stability of the mean of the true score of the resilience across time.

The standardized correlation (Pearson’s correlation) between the latent state factors were t1 with t2 = 0.791, *p* < 0.001, t1 with t3 = 0.696, *p* < 0.001, and t2 with t3 = 0.671, *p* < 0.001. The correlation of 0.671 corresponds to approximately 45.02% of shared variance (from 0.671^2^). This indicates that the construct of resilience under the CD-RISC structure is constituted by both trait and state. The highest correlation, 0.79, when squared, returns the value of 0.6241, meaning that 62.41% of the variance is due to trait.

Table 2 shows the correlation matrix between the latent state factors and IS factors for CD-RISC considering item 1 (on top) and item 4 (on bottom) as reference indicators. The correlation between IS factors “…represent[ed] stable variance in the non-reference parcels that is not shared with the reference parcel” (see [36] p. 139). Note that the LS-IS assumes that latent state factors and IS residual factors are orthogonal (i.e., zero correlation between them).

### RSA stability and change across time

As observed with the CD-RISC, the most restricted invariance level was also achieved for the RSA (see model 7), where the LS factors covariances were restricted to be equal. However, this interpretation is not so straight forward due to the discrepancy between the Satorra-Bentler chi-square significant (*p* = 0.0430), which could be interpreted as the most restricted invariance level was not achieved. On the other hand, we have values of AIC and BIC reducing, indicating that an improvement in the model’s fit given the added restriction. We considered the significance of the Satorra-Bentler chi-square significant negligible, because model 6 showed that the following correlation patterns between the state factors are proximal in terms of the magnitude: t1 with t2 (*r* = 0.803, *p*-value < 0.001), t1 with t3 (*r* = 0.816, *p* < 0.001), and t1 with t2 (*r* = 0.750, *p*-value < 0.001), indicating that resilience captured by the RSA parcels is also a trait-state construct. Such interpretation comes from the highest correlation, *r* = 0.816, when squared, corresponds to 0.6658 or 66.58% of trait variance, which, as described above, is not strong evidence of trait-like components being present. Therefore, model 7 shows that individuals average true score in the reference parcel (i.e., personal strength/perception of self) did not change across time and that the true individual differences in the reference parcel also remained constant across time. This indicates that there was no mean change of resilience true score across the three assessments. Moreover, Table 3 shows the correlation matrix between the latent state factors and IS factors for RSA. The standardized correlation between the latent state factors (*r* = 0.790, *p* < 0.001) is equal because RSA model 7 imposed such a restriction. The correlations between the parcel-specific residual factors refer to stable correlations between parcel-effects not shared with the first parcel.

**Table 3:** RSA correlation matrix between latent state factors and IS factors on top parcel A as reference and on bottom B

	T1	T2	T3	TB	TC	TD	TE	TF
T1	1.000							
T2	0.790	1.000						
T3	0.790	0.790	1.000					
TB	0.000	0.000	0.000	1.000				
TC	0.000	0.000	0.000	0.326	1.000			
TD	0.000	0.000	0.000	-0.095	0.042	1.000		
TE	0.000	0.000	0.000	0.122	0.185	0.153	1.000	
TF	0.000	0.000	0.000	0.150	0.243	0.445	0.640	1.000
	T1	T2	T3	TA	TC	TD	TE	TF
T1	1.000							
T2	0.738	1.000						
T3	0.738	0.738	1.000					
TA	0.000	0.000	0.000	1.000				
TC	0.000	0.000	0.000	-0.003	1.000			
TD	0.000	0.000	0.000	0.386	0.060	1.000		
TE	0.000	0.000	0.000	0.201	0.161	0.221	1.000	
TF	0.000	0.000	0.000	0.177	0.206	0.492	0.646	1.000

*Note*. T1 = state factor occasion 1, T2 = state factor occasion 2, T3 = state factor occasion 3, TA = Personal strength/Perception of self, TB = Personal strength/Perception of future, TC = Structured style, TD = Social competence, TE = Family cohesion, TF = Social resources

Therefore, it might be seen that the correlation between E and F do not share much information with the reference parcel, whereas D with B and D with C share more information with the reference parcel. Given that the reference parcel was arbitrarily chosen, a sensitivity analysis was run using parcel B (Personal strength/Perception of future) as a reference (bottom part of Table 3).

To summarize, both the CD-RISC and the RSA reached unique invariance level, which is a more than necessary level of invariance to allow comparisons of mean scores of resilience across time. In terms of trait-like and state-like spectrums of assessment, the closer the correlation between the state factors are to 1, the more trait-like the construct is. Given that a correlation between two state latent factors of 0.7071 corresponds to 50% shared variance (e.g., meaning half trait and half state are measured), the results (e.g., latent state factors correlations not higher than 0.81), suggest that there are both state and trait components in both resilience measures.

### Reliability

Tables 4 and 5 show the CD-RISC and RSA item reliabilities across time, respectively; one might call to attention that the items 1, 8 and 10 showed reliabilities below 0.70 across all the waves. For the RSA, the parcels were shown to have reliabilities above 0.70 across all occasions.

**Table 4:** Reliability (in R^2^) for CD-RISC across the three time points derived from LS-IS model

Time	Item	Reliability	Standard Error
1	Adaptation to changes	0.666	0.050
1	Coping with adversity	0.819	0.040
1	Humour	0.791	0.042
1	Coping with stress	0.747	0.046
1	Bouncing back	0.763	0.047
1	Achieving goals	0.775	0.041
1	Coping with pressure	0.784	0.044
1	Discouragement by failure	0.604	0.065
1	Strength during adversity	0.791	0.041
1	Handling feelings	0.694	0.049
2	Adaptation to changes	0.666	0.050
2	Coping with adversity	0.825	0.036
2	Humour	0.775	0.047
2	Coping with stress	0.677	0.056
2	Bouncing back	0.738	0.051
2	Achieving goals	0.769	0.041
2	Coping with pressure	0.733	0.053
2	Discouragement by failure	0.587	0.068
2	Strength during adversity	0.810	0.040
2	Handling feelings	0.640	0.050
3	Adaptation to changes	0.666	0.050
3	Coping with adversity	0.827	0.037
3	Humour	0.739	0.047
3	Coping with stress	0.608	0.056
3	Bouncing back	0.700	0.054
3	Achieving goals	0.692	0.056
3	Coping with pressure	0.714	0.058
3	Discouragement by failure	0.529	0.059
3	Strength during adversity	0.775	0.041
3	Handling feelings	0.646	0.047

**Table 5:** Reliabilities for RSA parcels across the three time points derived from LS-IS model

Time	Parcel	Reliability	Standard Error
1	Personal strength/Perception of self	0.845	0.032
2	Personal strength/Perception of self	0.845	0.032
3	Personal strength/Perception of self	0.845	0.032
1	Personal strength/Perception of future	0.716	0.039
2	Personal strength/Perception of future	0.716	0.039
3	Personal strength/Perception of future	0.716	0.039
1	Structured style	0.750	0.036
2	Structured style	0.750	0.036
3	Structured style	0.750	0.036
1	Social competence	0.839	0.021
2	Social competence	0.839	0.021
3	Social competence	0.839	0.021
1	Family cohesion	0.808	0.026
2	Family cohesion	0.808	0.026
3	Family cohesion	0.808	0.026
1	Social resources	0.797	0.028
2	Social resources	0.797	0.028
3	Social resources	0.797	0.028

### Missingness

The most common missing pattern was the monotonic one, where those participants missing at the second occasion were also missing at the third occasion. We found a lack of evidence regarding missingness being related to gender at the second (χ^2^(1) = 1.195, *p*-value = 0.274) and third occasion (χ^2^(1) = 1.389, *p*-value = 0.239). In terms of age, we found evidence for age, where older individuals were less likely to dropout at the second occasion (Odds Ratio = 0.966, 95% CI [0.950 to 0.982]), and the third occasion (Odds Ratio = 0.963, 95%CI [0.947 to 0.979]).

## Discussion

In this paper, we have applied a LS and LS-IS model to the CD-RISC [23] and the RSA [24] by evaluating their longitudinal measurement invariance. Both scales intend to measure resilience, and longitudinal invariance is a fundamental prerequisite to discuss stability, change, and the nature of the constructs in terms of being more trait-like or state-like. Both the CD-RISC and the RSA achieved measurement invariance at means, variances, and covariance between the latent state factors meaning that a) individuals’ average true resilience scores did not change across time, b) that the true individual differences in resilience also remained constant across time, and c) the correlation between the state factors were a strong indication of more trait-like constructs. However, caution should be taken in terms of the meaning of the state factor for both scales because for the RSA the correlations between the state variables refer to the true score correlations of the reference indicator (parcels), whereas the CD-RISC modeling is specified at item-level. Therefore, for the RSA, the state factor parcels, which are already a composite of the participants answers (subscale score) as indicators and for the CD-RISC, the questions (item-level). For the RSA, the parcels were used because the sample size was not large enough to model at item level. In line with such a rationale, we cannot compare the reliability directly for both scales because parcels due to their composite, have higher reliability. Therefore, we cannot directly say that the RSA had better reliability than the CD-RISC given the observed indicators under evaluation are different (parcel vs. items).

Our models suggest that while subscales of the RSA and items of the CD-RISC do capture trait-like components of resilience, there is a large amount of the variance that is occasion-specific (state). This suggests resilience may well be a complex process, which is difficult to operationalize via psychometric scales. In terms of psychometrics, the notion of more trait-like or state-like constructs are only possible to be evaluated under longitudinal design. Therefore, to examine such an issue, at least three occasions are necessary to separate both effects. Also, it is important to consider the effects obtained are conditioned to the time-lag between the occasions (three months) and for RSA we analyzed the parcels (not the 33 items). Given the RSA contains many items and our reduced sample size to model at item-level longitudinally, we opted to use parcels. This is therefore a limitation for the RSA model. In addition, the results should be generalized with caution as we have modelled a 3-month period only. In general, even questions designed to assess momentaneous behavior such as ‘right now, how do you feel’, are theoretically capturing more state components and are not guaranteed to assess 100% state variance (see [41]). Therefore, this could be the case for both statements included in the CD-RISC and the RSA because of the way they are worded. Perhaps for this study, the question is more: what are the scales measuring rather than asking, is resilience as a concept, a trait, or a process? According to our analysis, the two resilience scales utilized in this study (the CD-RISC and the RSA) contain trait-like and state-like components of resilience. A more in-depth understanding of the content of the two scales is needed to understand which ‘parts’ of resilience the scales are measuring, especially using models as state-trait models.

Ever since resilience was first introduced as a concept, there has been debate in the literature about whether resilience should be considered a stable character trait or a dynamic, changeable process which varies across time. Some studies have found support for resilience as a trait (e.g. [16, 17]). Although some authors in the field of resilience research have argued that resilience is a developmental process (state) which is not static, fixed or immutable [3, 10–12, 14], discussions have been theoretical and there has been limited evidence-based research. To our knowledge, no researcher has attempted to break down and disentangle the trait and state components of resilience scales using our methods. There are many processes and attributes involved in resilience so perhaps resilience should not be confined to a single trait or process but be understood in terms of a combination of both, as suggested by Masten and Obradović [42]. For example, Leys et al. [43] use social competence as an example of when components of resilience could be considered beneficial to the individual as either a trait or state. Moreover, the RSA includes social competence as a factor of resilience (formed of six items about being flexible in social situations, friendships and meeting new people) and in this study social competence was found to be more trait-based than state-based in employees. Perhaps some aspects of resilience (e.g. social competence) could be defined as a trait in the way that individuals are able to establish quality relationships, but it may also become more situational where having a present and supportive family could be both a help and a hindrance in certain situations [43]. Future studies could delve deeper into our understanding of specific factors of resilience to understand what aspects of resilience are advantageous in which situations (e.g. social competence as a trait vs. social competence as a state).

Researchers are often tempted to take a binary approach with resilience and report either a presence or absence of resilience in individuals [15], but most psychological constructs are neither completely trait-like or completely state-like (See [30]). To understand and measure resilience reliably, it is important that researchers outline what definition of resilience they are aligning their research with (e.g., resilience as a trait or state or both). In addition, researchers should be careful to check whether the measures they plan to use in their research actually measure resilience according to how they define resilience in their work. This is difficult with the discrepancies in the definition of resilience still under scrutiny and the limitations of self-report measures.

### Limitations

Resilience is difficult to measure objectively because of the complex processes it involves and conceptual discrepancies in the definition [44, 45]. For resilience, perhaps one of the biggest issues with self-report measures is their inability to capture the emotional components of resilience [43], which could be more state based. Measures of resilience are mainly based upon cognitive behavioral components of resilience and have been suggested to only be an indirect measure of the consequences of emotional processes as the processes behind resilience are only partially understood [43]. In addition, although there are theoretically sound arguments present within the literature as to why resilience should be considered a trait or a state, it is not clear what resilience scales are measuring. Resilience is usually inferred by measurement of the two main components of resilience: stress and competence [46]. Most resilience scales utilized in psychological research are based upon the presence of resources and characteristics or favorable mental health factors associated with resilience, rather than viewing resilience as an outcome [47, 48]. Therefore, this evidence questions whether resilience as a state a quantifiable concept is, or whether the concept is simply too abstract to be confined to a psychometric scale. The scales may just be measuring factors which could foster resilience or characteristics of resilience, rather than quantifying resilience itself. It is imperative that we continue research to understand if resilience scales are ecologically valid based on their theoretical basis and definition of resilience. In addition, this information is helpful to highlight for future researchers to consider the content of the scale used to measure resilience and whether those scales assess resilience in the form appropriate to their research.

As a possible limitation, the sample size was relatively small for modelling latent-state at item-level without avoiding the use of parcels for the RSA. So, conclusions at parcel-level (for the RSA) and at item-level (for the CD-RISC) are not comparable. Moreover, to methodologically understand change, stability, and the nature of the constructs in terms of being more trait-like or state-like, one must use longitudinal data and different models are available not only to disentangle states and traits, but also other statistical features of psychological constructs such as auto-regressive effects (aka., carryover effects) [34]. Moreover, depending on the lag between the repeated measures, the results described here also could change and there is not a timeframe cutoff to describe how distance in time between the repeated measures in both scales should be conducted.

Other possible limitations with regards to the sample were that the participants were relatively young (M age = 34.05 years). The office of national statistics reported in 2019 (ONS, 2019) that the age group 35 to 49 years old had the highest employment rates (85.2%), closely followed by those aged 25 to 34 years old (84.4%). In addition, the black, Asian and minority ethnic community were insufficiently represented in the sample as most of the participants were White British. Improved effort should be made in the future to gain a more representative sample of the desired population according to age and ethnicity, as this could affect elements of resilience. However, the scales we used were appropriate for the population we sampled. To our knowledge this LST theory and their related models have not been used before with these resilience measures and this research adds to our understanding of how such a construct underlying the CD-RISC and the RSA behaves in terms of stability and change across time.

### Recommendations and take home message

The authors of this paper recommend the use of both the CD-RISC and the RSA for adult populations (in line with Smith-Osborne & Whitehill Bolton [18]), depending on the authors interpretation of resilience and what they want to measure. Perhaps if researchers are more interested in overall ability to withstand stress despite challenges, the CD-RISC would be more appropriate due to the content of the scale. On the other hand, if the research question relates to the different strengths of an individual which could foster resilience and ability to cope with stress, the RSA could be more suited (e.g., subscales including social competence and family cohesion). The scales have good psychometric properties and are a useful tool for quantifying resilience (as much as we can quantify an abstract construct in a psychometric scale).

Knowing whether a construct carries more trait or state-like features brings new perspectives in the analysis of trajectories for resilience behaviors under a fine-grained lens of psychometrics. Disentangling such sources of information allows, under experimental design, to answer questions such as ‘are traits or states changing?’, which is not answerable by regular longitudinal techniques such as generalized estimation equations and mixed effect models. This paper has provided some unique insight into what resilience scales are measuring (trait or state) and highlighted the complexity of the phenomenon.

## Conclusions

In conclusion, our models suggested that both the RSA [24] and the CD-RISC [23] capture trait-like components but there is still a large amount of variance that is occasion-specific (state). The LSM and LS-IS models used in this study are fundamental in understanding stability, change and the trait and state-like nature of constructs such as resilience. The findings highlight the importance of understanding the content of resilience scales and why researchers should be aware of what resilience scales are capturing when utilizing them in research. This study highlights the need for further investigation of resilience scales and identification of state and trait-based components. Resilience is a complex phenomenon and is therefore difficult to operationalize with self-report measures as these measures do not assess the emotional components of resilience.

## Supplementary Information

**Additional file 1:** CD-RISC modelling syntaxes for Mplus.

**Additional file 2:** RSA modelling syntaxes for Mplus.

## Data Availability

The datasets generated and analysed during the current study are not publicly available due to confidentiality of employee’s records, but statistical analyses are available from the corresponding author on reasonable request.
