# Supplementary material for: Disentangling change across the time and true stability of employees’ resilience using latent state model
Source: BMC Psychiatry. 2022 Oct 20;22:651. doi: 10.1186/s12888-022-04294-3 (PMC9583564; doi:10.1186/s12888-022-04294-3)
Supplement: Supplementary file 1 — Additional file 1: CD-RISC modelling syntaxes for Mplus. [file 12888_2022_4294_MOESM1_ESM.docx]

CD-RISC

**Configural model**

usevariable are T1_1 T1_2 T1_3 T1_4 T1_5 T1_6

T1_7 T1_8 T1_9 T1_10 T2_1 T2_2 T2_3 T2_4 T2_5 T2_6 T2_7 T2_8 T2_9 T2_10

T3_1 T3_2 T3_3 T3_4 T3_5 T3_6 T3_7 T3_8 T3_9 T3_10;

categorical are T1_1 T1_2 T1_3 T1_4 T1_5 T1_6

T1_7 T1_8 T1_9 T1_10 T2_1 T2_2 T2_3 T2_4 T2_5 T2_6 T2_7 T2_8 T2_9 T2_10

T3_1 T3_2 T3_3 T3_4 T3_5 T3_6 T3_7 T3_8 T3_9 T3_10;

missing are all (-99);

ANALYSIS:

PARAMETERIZATION = THETA;

ITERATIONS=3000;

ESTIMATOR=WLSMV;

Model:

!Free factor loadings on the state latent factors!

TAU1 by T1_1*

T1_2@1

T1_3*

T1_4*

T1_5*

T1_6*

T1_7*

T1_8*

T1_9*

T1_10*;

TAU2 BY T2_1*

T2_2@1

T2_3*

T2_4*

T2_5*

T2_6*

T2_7*

T2_8*

T2_9*

T2_10*;

TAU3 BY T3_1*

T3_2@1

T3_3*

T3_4*

T3_5*

T3_6*

T3_7*

T3_8*

T3_9*

T3_10*;

!item specific residual factor!

TB by T1_2 T2_2 T3_2;

TC by T1_3 T2_3 T3_3;

TD by T1_4 T2_4 T3_4;

TE by T1_5 T2_5 T3_5;

TF by T1_6 T2_6 T3_6;

TG by T1_7 T2_7 T3_7;

TH by T1_8 T2_8 T3_8;

TI by T1_9 T2_9 T3_9;

TJ by T1_10 T2_10 T3_10;

!covariance between the item-specific residual factors; covariance between the state factors; orthogonality between state and item-specific factors!

TB-TJ WITH TAU1-TAU3@0;

! First thresholds of the first item equal across the three time points!

[T1_1$1 T2_1$1 T3_1$1](11);

[T1_1$2 T2_1$2 T3_1$2];

[T1_1$3 T2_1$3 T3_1$3];

! First thresholds of the second item equal across the three time points!

[T1_2$1 T2_2$1 T3_2$1](1);

! Second thresholds of the second item equal across the three time points!

[T1_2$2 T2_2$2 T3_2$2](2);

[T1_2$3 T2_2$3 T3_2$3];

! First thresholds of the third item equal across the three time points!

[T1_3$1 T2_3$1 T3_3$1](3);

[T1_3$2 T2_3$2 T3_3$2];

[T1_3$3 T2_3$3 T3_3$3];

! First thresholds of the fourth item equal across the three time points!

[T1_4$1 T2_4$1 T3_4$1](4);

[T1_4$2 T2_4$2 T3_4$2];

[T1_4$3 T2_4$3 T3_4$3];

! First thresholds of the fifth item equal across the three time points!

[T1_5$1 T2_5$1 T3_5$1](5);

[T1_5$2 T2_5$2 T3_5$2];

[T1_5$3 T2_5$3 T3_5$3];

! First thresholds of the sixth item equal across the three time points!

[T1_6$1 T2_6$1 T3_6$1](6);

[T1_6$2 T2_6$2 T3_6$2];

[T1_6$3 T2_6$3 T3_6$3]; !

First thresholds of the seventh item equal across the three time points!

[T1_7$1 T2_7$1 T3_7$1](7);

[T1_7$2 T2_7$2 T3_7$2];

[T1_7$3 T2_7$3 T3_7$3];

! First thresholds of the eighth item equal across the three time points!

[T1_8$1 T2_8$1 T3_8$1](8);

[T1_8$2 T2_8$2 T3_8$2];

[T1_8$3 T2_8$3 T3_8$3];

! First thresholds of the nineth item equal across the three time points!

[T1_9$1 T2_9$1 T3_9$1](9);

[T1_9$2 T2_9$2 T3_9$2];

[T1_9$3 T2_9$3 T3_9$3];

! First thresholds of the tenth item equal across the three time points!

[T1_10$1 T2_10$1 T3_10$1](10);

[T1_10$2 T2_10$2 T3_10$2];

[T1_10$3 T2_10$3 T3_10$3];

! Common factor covariance matrix;!

TAU1-TAU3 WITH TAU1-TAU3;

! Common factor means; first factor mean fixed at zero as refence!

[TAU1@0];

[TAU2*];

[TAU3*];

! Unique variances --- residual variances for al the first wave indicators fixed at zero!

T1_1@1 T1_2@1 T1_3@1 T1_4@1 T1_5@1 T1_6@1 T1_7@1 T1_8@1 T1_9@1 T1_10@1

T2_1 T2_2 T2_3 T2_4 T2_5 T2_6 T2_7 T2_8 T2_9 T2_10

T3_1 T3_2 T3_3 T3_4 T3_5 T3_6 T3_7 T3_8 T3_9 T3_10;

SAVEDATA: DIFFTEST IS baseline.dat;

OUTPUT: sampstat residual mod(all 10) standardized;

**!!Weak invariance**

!factor loading on the state continuous latent factors are held constant across time!

TAU1 by T1_1* (a)

T1_2@1 (b)

T1_3* (c)

T1_4* (d)

T1_5* (e)

T1_6* (f)

T1_7* (g)

T1_8* (h)

T1_9* (i)

T1_10* (j);

TAU2 BY T2_1* (a)

T2_2@1 (b)

T2_3* (c)

T2_4* (d)

T2_5* (e)

T2_6* (f)

T2_7* (g)

T2_8* (h)

T2_9* (i)

T2_10* (j);

TAU3 BY T3_1* (a)

T3_2@1 (b)

T3_3* (c)

T3_4* (d)

T3_5* (e)

T3_6* (f)

T3_7* (g)

T3_8* (h)

T3_9* (i)

T3_10* (j);

All the other part of the syntax are equal to the configural model

SAVEDATA: DIFFTEST IS loading.dat;

OUTPUT: sampstat residual mod(all 10) standardized;

**Strong invariance**

ANALYSIS:

PARAMETERIZATION = THETA;

ITERATIONS=3000;

ESTIMATOR=WLSMV;

DIFFTEST = loading.DAT;

Model:

! !factor loading on the state continuous latent factors are held constant across time!

TAU1 by T1_1* (a)

T1_2@1 (b)

T1_3* (c)

T1_4* (d)

T1_5* (e)

T1_6* (f)

T1_7* (g)

T1_8* (h)

T1_9* (i)

T1_10* (j);

TAU2 BY T2_1* (a)

T2_2@1 (b)

T2_3* (c)

T2_4* (d)

T2_5* (e)

T2_6* (f)

T2_7* (g)

T2_8* (h)

T2_9* (i)

T2_10* (j);

TAU3 BY T3_1* (a)

T3_2@1 (b)

T3_3* (c)

T3_4* (d)

T3_5* (e)

T3_6* (f)

T3_7* (g)

T3_8* (h)

T3_9* (i)

T3_10* (j);

TB by T1_2 T2_2 T3_2;

TC by T1_3 T2_3 T3_3;

TD by T1_4 T2_4 T3_4;

TE by T1_5 T2_5 T3_5;

TF by T1_6 T2_6 T3_6;

TG by T1_7 T2_7 T3_7;

TH by T1_8 T2_8 T3_8;

TI by T1_9 T2_9 T3_9;

TJ by T1_10 T2_10 T3_10;

TB-TJ WITH TAU1-TAU3@0;

[T1_1$1 T2_1$1 T3_1$1](11);

[T1_1$2 T2_1$2 T3_1$2](12);

[T1_1$3 T2_1$3 T3_1$3](13);

[T1_2$1 T2_2$1 T3_2$1](1);

[T1_2$2 T2_2$2 T3_2$2](2);

[T1_2$3 T2_2$3 T3_2$3](14);

[T1_3$1 T2_3$1 T3_3$1](3);

[T1_3$2 T2_3$2 T3_3$2](15);

[T1_3$3 T2_3$3 T3_3$3](16);

[T1_4$1 T2_4$1 T3_4$1](4);

[T1_4$2 T2_4$2 T3_4$2](17);

[T1_4$3 T2_4$3 T3_4$3](18);

[T1_5$1 T2_5$1 T3_5$1](5);

[T1_5$2 T2_5$2 T3_5$2](19);

[T1_5$3 T2_5$3 T3_5$3](20);

[T1_6$1 T2_6$1 T3_6$1](6);

[T1_6$2 T2_6$2 T3_6$2](21);

[T1_6$3 T2_6$3 T3_6$3](22);

[T1_7$1 T2_7$1 T3_7$1](7);

[T1_7$2 T2_7$2 T3_7$2](23);

[T1_7$3 T2_7$3 T3_7$3](24);

[T1_8$1 T2_8$1 T3_8$1](8);

[T1_8$2 T2_8$2 T3_8$2](25);

[T1_8$3 T2_8$3 T3_8$3](26);

[T1_9$1 T2_9$1 T3_9$1](9);

[T1_9$2 T2_9$2 T3_9$2](27);

[T1_9$3 T2_9$3 T3_9$3](28);

[T1_10$1 T2_10$1 T3_10$1](10);

[T1_10$2 T2_10$2 T3_10$2](29);

[T1_10$3 T2_10$3 T3_10$3](30);

!Common factor covariance matrix;!

TAU1-TAU3 WITH TAU1-TAU3;

! Common factor means;!

[TAU1@0];

[TAU2*];

[TAU3*];

! Unique variances;!

T1_1@1 T1_2@1 T1_3@1 T1_4@1 T1_5@1 T1_6@1 T1_7@1 T1_8@1 T1_9@1 T1_10@1

T2_1 T2_2 T2_3 T2_4 T2_5 T2_6 T2_7 T2_8 T2_9 T2_10

T3_1 T3_2 T3_3 T3_4 T3_5 T3_6 T3_7 T3_8 T3_9 T3_10;

SAVEDATA: DIFFTEST IS threshold.dat;

**Residual invariance model**

ANALYSIS:

PARAMETERIZATION = THETA;

ITERATIONS=3000;

ESTIMATOR=WLSMV;

DIFFTEST = threshold.DAT;

Model:

TAU1 by T1_1* (a)

T1_2@1 (b)

T1_3* (c)

T1_4* (d)

T1_5* (e)

T1_6* (f)

T1_7* (g)

T1_8* (h)

T1_9* (i)

T1_10* (j);

TAU2 BY T2_1* (a)

T2_2@1 (b)

T2_3* (c)

T2_4* (d)

T2_5* (e)

T2_6* (f)

T2_7* (g)

T2_8* (h)

T2_9* (i)

T2_10* (j);

TAU3 BY T3_1* (a)

T3_2@1 (b)

T3_3* (c)

T3_4* (d)

T3_5* (e)

T3_6* (f)

T3_7* (g)

T3_8* (h)

T3_9* (i)

T3_10* (j);

TB by T1_2 T2_2 T3_2;

TC by T1_3 T2_3 T3_3;

TD by T1_4 T2_4 T3_4;

TE by T1_5 T2_5 T3_5;

TF by T1_6 T2_6 T3_6;

TG by T1_7 T2_7 T3_7;

TH by T1_8 T2_8 T3_8;

TI by T1_9 T2_9 T3_9;

TJ by T1_10 T2_10 T3_10;

TB-TJ WITH TAU1-TAU3@0;

[T1_1$1 T2_1$1 T3_1$1](11);

[T1_1$2 T2_1$2 T3_1$2](12);

[T1_1$3 T2_1$3 T3_1$3](13);

[T1_2$1 T2_2$1 T3_2$1](1);

[T1_2$2 T2_2$2 T3_2$2](2);

[T1_2$3 T2_2$3 T3_2$3](14);

[T1_3$1 T2_3$1 T3_3$1](3);

[T1_3$2 T2_3$2 T3_3$2](15);

[T1_3$3 T2_3$3 T3_3$3](16);

[T1_4$1 T2_4$1 T3_4$1](4);

[T1_4$2 T2_4$2 T3_4$2](17);

[T1_4$3 T2_4$3 T3_4$3](18);

[T1_5$1 T2_5$1 T3_5$1](5);

[T1_5$2 T2_5$2 T3_5$2](19);

[T1_5$3 T2_5$3 T3_5$3](20);

[T1_6$1 T2_6$1 T3_6$1](6);

[T1_6$2 T2_6$2 T3_6$2](21);

[T1_6$3 T2_6$3 T3_6$3](22);

[T1_7$1 T2_7$1 T3_7$1](7);

[T1_7$2 T2_7$2 T3_7$2](23);

[T1_7$3 T2_7$3 T3_7$3](24);

[T1_8$1 T2_8$1 T3_8$1](8);

[T1_8$2 T2_8$2 T3_8$2](25);

[T1_8$3 T2_8$3 T3_8$3](26);

[T1_9$1 T2_9$1 T3_9$1](9);

[T1_9$2 T2_9$2 T3_9$2](27);

[T1_9$3 T2_9$3 T3_9$3](28);

[T1_10$1 T2_10$1 T3_10$1](10);

[T1_10$2 T2_10$2 T3_10$2](29);

[T1_10$3 T2_10$3 T3_10$3](30);

! Common factor covariance matrix;!

TAU1-TAU3 WITH TAU1-TAU3;

! Common factor means;!

[TAU1@0];

[TAU2*];

[TAU3*];

! Unique variances; all set to one!

T1_1@1 T1_2@1 T1_3@1 T1_4@1 T1_5@1 T1_6@1 T1_7@1 T1_8@1 T1_9@1 T1_10@1

T2_1@1 T2_2@1 T2_3@1 T2_4@1 T2_5@1 T2_6@1 T2_7@1 T2_8@1 T2_9@1 T2_10@1

T3_1@1 T3_2@1 T3_3@1 T3_4@1 T3_5@1 T3_6@1 T3_7@1 T3_8@1 T3_9@1 T3_10@1;

SAVEDATA: DIFFTEST IS unique.dat;

**Model where the correlation between the state latent factor set to the same;**

ANALYSIS:

PARAMETERIZATION = THETA;

ITERATIONS=3000;

ESTIMATOR=WLSMV;

DIFFTEST = unique.DAT;

Model:

TAU1 by T1_1* (a)

T1_2@1 (b)

T1_3* (c)

T1_4* (d)

T1_5* (e)

T1_6* (f)

T1_7* (g)

T1_8* (h)

T1_9* (i)

T1_10* (j);

TAU2 BY T2_1* (a)

T2_2@1 (b)

T2_3* (c)

T2_4* (d)

T2_5* (e)

T2_6* (f)

T2_7* (g)

T2_8* (h)

T2_9* (i)

T2_10* (j);

TAU3 BY T3_1* (a)

T3_2@1 (b)

T3_3* (c)

T3_4* (d)

T3_5* (e)

T3_6* (f)

T3_7* (g)

T3_8* (h)

T3_9* (i)

T3_10* (j);

TB by T1_2 T2_2 T3_2;

TC by T1_3 T2_3 T3_3;

TD by T1_4 T2_4 T3_4;

TE by T1_5 T2_5 T3_5;

TF by T1_6 T2_6 T3_6;

TG by T1_7 T2_7 T3_7;

TH by T1_8 T2_8 T3_8;

TI by T1_9 T2_9 T3_9;

TJ by T1_10 T2_10 T3_10;

TB-TJ WITH TAU1-TAU3@0;

[T1_1$1 T2_1$1 T3_1$1](11);

[T1_1$2 T2_1$2 T3_1$2](12);

[T1_1$3 T2_1$3 T3_1$3](13);

[T1_2$1 T2_2$1 T3_2$1](1);

[T1_2$2 T2_2$2 T3_2$2](2);

[T1_2$3 T2_2$3 T3_2$3](14);

[T1_3$1 T2_3$1 T3_3$1](3);

[T1_3$2 T2_3$2 T3_3$2](15);

[T1_3$3 T2_3$3 T3_3$3](16);

[T1_4$1 T2_4$1 T3_4$1](4);

[T1_4$2 T2_4$2 T3_4$2](17);

[T1_4$3 T2_4$3 T3_4$3](18);

[T1_5$1 T2_5$1 T3_5$1](5);

[T1_5$2 T2_5$2 T3_5$2](19);

[T1_5$3 T2_5$3 T3_5$3](20);

[T1_6$1 T2_6$1 T3_6$1](6);

[T1_6$2 T2_6$2 T3_6$2](21);

[T1_6$3 T2_6$3 T3_6$3](22);

[T1_7$1 T2_7$1 T3_7$1](7);

[T1_7$2 T2_7$2 T3_7$2](23);

[T1_7$3 T2_7$3 T3_7$3](24);

[T1_8$1 T2_8$1 T3_8$1](8);

[T1_8$2 T2_8$2 T3_8$2](25);

[T1_8$3 T2_8$3 T3_8$3](26);

[T1_9$1 T2_9$1 T3_9$1](9);

[T1_9$2 T2_9$2 T3_9$2](27);

[T1_9$3 T2_9$3 T3_9$3](28);

[T1_10$1 T2_10$1 T3_10$1](10);

[T1_10$2 T2_10$2 T3_10$2](29);

[T1_10$3 T2_10$3 T3_10$3](30);

! Common factor covariance matrix;!

TAU1-TAU3 WITH TAU1-TAU3 (corr);

! Common factor means;!

[TAU1@0];

[TAU2*];

[TAU3*];

! Unique variances;!

T1_1@1 T1_2@1 T1_3@1 T1_4@1 T1_5@1 T1_6@1 T1_7@1 T1_8@1 T1_9@1 T1_10@1

T2_1@1 T2_2@1 T2_3@1 T2_4@1 T2_5@1 T2_6@1 T2_7@1 T2_8@1 T2_9@1 T2_10@1

T3_1@1 T3_2@1 T3_3@1 T3_4@1 T3_5@1 T3_6@1 T3_7@1 T3_8@1 T3_9@1 T3_10@1;

SAVEDATA: DIFFTEST IS correl.dat;

**Model with the means of the state latent factors set as equal;**

ANALYSIS:

PARAMETERIZATION = THETA;

ITERATIONS=3000;

ESTIMATOR=WLSMV;

DIFFTEST = correl.DAT;

Model:

TAU1 by T1_1* (a)

T1_2@1 (b)

T1_3* (c)

T1_4* (d)

T1_5* (e)

T1_6* (f)

T1_7* (g)

T1_8* (h)

T1_9* (i)

T1_10* (j);

TAU2 BY T2_1* (a)

T2_2@1 (b)

T2_3* (c)

T2_4* (d)

T2_5* (e)

T2_6* (f)

T2_7* (g)

T2_8* (h)

T2_9* (i)

T2_10* (j);

TAU3 BY T3_1* (a)

T3_2@1 (b)

T3_3* (c)

T3_4* (d)

T3_5* (e)

T3_6* (f)

T3_7* (g)

T3_8* (h)

T3_9* (i)

T3_10* (j);

TA by T1_1 T2_1 T3_1;

TB by T1_2 T2_2 T3_2;

TC by T1_3 T2_3 T3_3;

!TD by T1_4 T2_4 T3_4;

TE by T1_5 T2_5 T3_5;

TF by T1_6 T2_6 T3_6;

TG by T1_7 T2_7 T3_7;

TH by T1_8 T2_8 T3_8;

TI by T1_9 T2_9 T3_9;

TJ by T1_10 T2_10 T3_10;

TA-TJ WITH TAU1-TAU3@0;

[T1_1$1 T2_1$1 T3_1$1](11);

[T1_1$2 T2_1$2 T3_1$2](12);

[T1_1$3 T2_1$3 T3_1$3](13);

[T1_2$1 T2_2$1 T3_2$1](1);

[T1_2$2 T2_2$2 T3_2$2](2);

[T1_2$3 T2_2$3 T3_2$3](14);

[T1_3$1 T2_3$1 T3_3$1](3);

[T1_3$2 T2_3$2 T3_3$2](15);

[T1_3$3 T2_3$3 T3_3$3](16);

[T1_4$1 T2_4$1 T3_4$1](4);

[T1_4$2 T2_4$2 T3_4$2](17);

[T1_4$3 T2_4$3 T3_4$3](18);

[T1_5$1 T2_5$1 T3_5$1](5);

[T1_5$2 T2_5$2 T3_5$2](19);

[T1_5$3 T2_5$3 T3_5$3](20);

[T1_6$1 T2_6$1 T3_6$1](6);

[T1_6$2 T2_6$2 T3_6$2](21);

[T1_6$3 T2_6$3 T3_6$3](22);

[T1_7$1 T2_7$1 T3_7$1](7);

[T1_7$2 T2_7$2 T3_7$2](23);

[T1_7$3 T2_7$3 T3_7$3](24);

[T1_8$1 T2_8$1 T3_8$1](8);

[T1_8$2 T2_8$2 T3_8$2](25);

[T1_8$3 T2_8$3 T3_8$3](26);

[T1_9$1 T2_9$1 T3_9$1](9);

[T1_9$2 T2_9$2 T3_9$2](27);

[T1_9$3 T2_9$3 T3_9$3](28);

[T1_10$1 T2_10$1 T3_10$1](10);

[T1_10$2 T2_10$2 T3_10$2](29);

[T1_10$3 T2_10$3 T3_10$3](30);

! Common factor covariance matrix;!

TAU1-TAU3 WITH TAU1-TAU3 (corr);

! Common factor means;!

[TAU1@0];

!Here means of state latent factor for time 2 and time 3 are set as equal!

[TAU2* TAU3*] (means);

! Unique variances;!

T1_1@1 T1_2@1 T1_3@1 T1_4@1 T1_5@1 T1_6@1 T1_7@1 T1_8@1 T1_9@1 T1_10@1

T2_1@1 T2_2@1 T2_3@1 T2_4@1 T2_5@1 T2_6@1 T2_7@1 T2_8@1 T2_9@1 T2_10@1

T3_1@1 T3_2@1 T3_3@1 T3_4@1 T3_5@1 T3_6@1 T3_7@1 T3_8@1 T3_9@1 T3_10@1;

SAVEDATA: DIFFTEST IS means.dat;

**Model where the common factor covariance matrix is set to be equal**

….all previous commands are also the same.

! here is the new amendment where the common factor covariance matrix is specified to be equal;!

TAU1-TAU3 WITH TAU1-TAU3 (corr);

! Common factor means;!

[TAU1@0];

[TAU2* TAU3*] (means);

! variances of the state latent factor set to be equal!

tau1-tau3 (var);

! Unique variances;!

T1_1@1 T1_2@1 T1_3@1 T1_4@1 T1_5@1 T1_6@1 T1_7@1 T1_8@1 T1_9@1 T1_10@1

T2_1@1 T2_2@1 T2_3@1 T2_4@1 T2_5@1 T2_6@1 T2_7@1 T2_8@1 T2_9@1 T2_10@1

T3_1@1 T3_2@1 T3_3@1 T3_4@1 T3_5@1 T3_6@1 T3_7@1 T3_8@1 T3_9@1 T3_10@1;
