# Supplementary material for: Disentangling change across the time and true stability of employees’ resilience using latent state model
Source: BMC Psychiatry. 2022 Oct 20;22:651. doi: 10.1186/s12888-022-04294-3 (PMC9583564; doi:10.1186/s12888-022-04294-3)
Supplement: Supplementary file 2 — Additional file 2: RSA modelling syntaxes for Mplus. [file 12888_2022_4294_MOESM2_ESM.docx]

!Configural model!

usevariable are

! Here we declare the six parcels, continuous observed indicators, of RSA!

T1_RSA_A_TOTAL T2_RSA_A_TOTAL T3_RSA_A_TOTAL

T1_RSA_B_TOTAL T2_RSA_B_TOTAL T3_RSA_B_TOTAL

T1_RSA_C_TOTAL T2_RSA_C_TOTAL T3_RSA_C_TOTAL

T1_RSA_D_TOTAL T2_RSA_D_TOTAL T3_RSA_D_TOTAL

T1_RSA_E_TOTAL T2_RSA_E_TOTAL T3_RSA_E_TOTAL

T1_RSA_F_TOTAL T2_RSA_F_TOTAL T3_RSA_F_TOTAl;

missing are all (-99);

Analysis:

ESTIMATOR = MLR;

Model:

!here tau1, tau2, tau3 are the state continuous variables!

Tau1 by

T1_RSA_A_TOTAL

T1_RSA_B_TOTAL

T1_RSA_C_TOTAL

T1_RSA_D_TOTAL

T1_RSA_E_TOTAL

T1_RSA_F_TOTAL ;

Tau2 by

T2_RSA_A_TOTAL

T2_RSA_B_TOTAL

T2_RSA_C_TOTAL

T2_RSA_D_TOTAL

T2_RSA_E_TOTAL

T2_RSA_F_TOTAL ;

Tau3 by

T3_RSA_A_TOTAL

T3_RSA_B_TOTAL

T3_RSA_C_TOTAL

T3_RSA_D_TOTAL

T3_RSA_E_TOTAL

T3_RSA_F_TOTAL ;

! Here, for identification purposes, we are fixing the first threshold for the first parcels across the three time points to be equal to zero!

[T1_RSA_A_TOTAL@0 T2_RSA_A_TOTAL@0 T3_RSA_A_TOTAL@0];

[T1_RSA_B_TOTAL T2_RSA_B_TOTAL T3_RSA_B_TOTAL];

[T1_RSA_C_TOTAL T2_RSA_C_TOTAL T3_RSA_C_TOTAL];

[T1_RSA_D_TOTAL T2_RSA_D_TOTAL T3_RSA_D_TOTAL];

[T1_RSA_E_TOTAL T2_RSA_E_TOTAL T3_RSA_E_TOTAL];

[T1_RSA_F_TOTAL T2_RSA_F_TOTAL T3_RSA_F_TOTAL];

!here we are declaring that the variances for the three latent variables are equal!

!t1 t2 t3 (Var_tau);

!here we are specifying that the mean of the three latent variables are free to be estimated!

[tau1* tau2* tau3*];

!here we specified the parcel specific latent indicators, using parcel A as reference – that’s why it is not cite here; all the parcels indicators are fixed at one!

TB by T1_RSA_B_TOTAL@1 T2_RSA_B_TOTAL@1 T3_RSA_B_TOTAL@1;

TC by T1_RSA_C_TOTAL@1 T2_RSA_C_TOTAL@1 T3_RSA_C_TOTAL@1;

TD by T1_RSA_D_TOTAL@1 T2_RSA_D_TOTAL@1 T3_RSA_D_TOTAL@1;

TE by T1_RSA_E_TOTAL@1 T2_RSA_E_TOTAL@1 T3_RSA_E_TOTAL@1;

TF by T1_RSA_F_TOTAL@1 T2_RSA_F_TOTAL@1 T3_RSA_F_TOTAL@1;

!here we specified that the parcel specific latent indicators are correlated with each other but are not correlated with the state latent indicators!

TB TC TD TE TF WITH Tau1@0 Tau2@0 Tau3@0;

SAVEDATA: DIFFTEST IS config.dat;

!weak model!

!Here we are specifying that the factor loadings for the same parcel are equal across the time!

Model:

T1 by

T1_RSA_A_TOTAL (a)

T1_RSA_B_TOTAL (b)

T1_RSA_C_TOTAL (c)

T1_RSA_D_TOTAL (d)

T1_RSA_E_TOTAL (e)

T1_RSA_F_TOTAL (f);

T2 by

T2_RSA_A_TOTAL (a)

T2_RSA_B_TOTAL (b)

T2_RSA_C_TOTAL (c)

T2_RSA_D_TOTAL (d)

T2_RSA_E_TOTAL (e)

T2_RSA_F_TOTAL (f);

T3 by

T3_RSA_A_TOTAL (a)

T3_RSA_B_TOTAL (b)

T3_RSA_C_TOTAL (c)

T3_RSA_D_TOTAL (d)

T3_RSA_E_TOTAL (e)

T3_RSA_F_TOTAL (f);

! from here all the constraints are the same as configural model!

[T1_RSA_A_TOTAL@0 T2_RSA_A_TOTAL@0 T3_RSA_A_TOTAL@0];

! the means of the state factor are free to be estimated across the time!

[t1* t2* t3*];

!here we specified the parcel specific latent indicators, using parcel A as reference – that’s why it is not cite here; all the parcels indicators are fixed at one!

TB by T1_RSA_B_TOTAL@1 T2_RSA_B_TOTAL@1 T3_RSA_B_TOTAL@1;

TC by T1_RSA_C_TOTAL@1 T2_RSA_C_TOTAL@1 T3_RSA_C_TOTAL@1;

TD by T1_RSA_D_TOTAL@1 T2_RSA_D_TOTAL@1 T3_RSA_D_TOTAL@1;

TE by T1_RSA_E_TOTAL@1 T2_RSA_E_TOTAL@1 T3_RSA_E_TOTAL@1;

TF by T1_RSA_F_TOTAL@1 T2_RSA_F_TOTAL@1 T3_RSA_F_TOTAL@1;

TB TC TD TE TF WITH T1@0 T2@0 T3@0;

SAVEDATA: DIFFTEST IS weak.dat;

!strong model!

! here factor loadings from the same parcel are constraint to be equal across the time!

Model:

T1 by

T1_RSA_A_TOTAL (a)

T1_RSA_B_TOTAL (b)

T1_RSA_C_TOTAL (c)

T1_RSA_D_TOTAL (d)

T1_RSA_E_TOTAL (e)

T1_RSA_F_TOTAL (f);

T2 by

T2_RSA_A_TOTAL (a)

T2_RSA_B_TOTAL (b)

T2_RSA_C_TOTAL (c)

T2_RSA_D_TOTAL (d)

T2_RSA_E_TOTAL (e)

T2_RSA_F_TOTAL (f);

T3 by

T3_RSA_A_TOTAL (a)

T3_RSA_B_TOTAL (b)

T3_RSA_C_TOTAL (c)

T3_RSA_D_TOTAL (d)

T3_RSA_E_TOTAL (e)

T3_RSA_F_TOTAL (f);

! here parcel As’ intercepts are fixed at zero across the three time points. For the other parcels they are hold as equal across the three time points!

[T1_RSA_A_TOTAL@0 T2_RSA_A_TOTAL@0 T3_RSA_A_TOTAL@0];

[T1_RSA_B_TOTAL T2_RSA_B_TOTAL T3_RSA_B_TOTAL](int2);

[T1_RSA_C_TOTAL T2_RSA_C_TOTAL T3_RSA_C_TOTAL](int3);

[T1_RSA_D_TOTAL T2_RSA_D_TOTAL T3_RSA_D_TOTAL](int4);

[T1_RSA_E_TOTAL T2_RSA_E_TOTAL T3_RSA_E_TOTAL](int5);

[T1_RSA_F_TOTAL T2_RSA_F_TOTAL T3_RSA_F_TOTAL](int6);

[t1* t2* t3*];

!here we specified the parcel specific latent indicators, using parcel A as reference – that’s why it is not cite here; all the parcels indicators are fixed at one!

TB by T1_RSA_B_TOTAL@1 T2_RSA_B_TOTAL@1 T3_RSA_B_TOTAL@1;

TC by T1_RSA_C_TOTAL@1 T2_RSA_C_TOTAL@1 T3_RSA_C_TOTAL@1;

TD by T1_RSA_D_TOTAL@1 T2_RSA_D_TOTAL@1 T3_RSA_D_TOTAL@1;

TE by T1_RSA_E_TOTAL@1 T2_RSA_E_TOTAL@1 T3_RSA_E_TOTAL@1;

TF by T1_RSA_F_TOTAL@1 T2_RSA_F_TOTAL@1 T3_RSA_F_TOTAL@1;

TB TC TD TE TF WITH T1@0 T2@0 T3@0;

SAVEDATA: DIFFTEST IS strong.dat;

!Strick model!

Analysis:

ESTIMATOR = MLR;

Difftest = strong.dat;

Model:

T1 by

T1_RSA_A_TOTAL (a)

T1_RSA_B_TOTAL (b)

T1_RSA_C_TOTAL (c)

T1_RSA_D_TOTAL (d)

T1_RSA_E_TOTAL (e)

T1_RSA_F_TOTAL (f);

T2 by

T2_RSA_A_TOTAL (a)

T2_RSA_B_TOTAL (b)

T2_RSA_C_TOTAL (c)

T2_RSA_D_TOTAL (d)

T2_RSA_E_TOTAL (e)

T2_RSA_F_TOTAL (f);

T3 by

T3_RSA_A_TOTAL (a)

T3_RSA_B_TOTAL (b)

T3_RSA_C_TOTAL (c)

T3_RSA_D_TOTAL (d)

T3_RSA_E_TOTAL (e)

T3_RSA_F_TOTAL (f);

[T1_RSA_A_TOTAL@0 T2_RSA_A_TOTAL@0 T3_RSA_A_TOTAL@0];

[T1_RSA_B_TOTAL T2_RSA_B_TOTAL T3_RSA_B_TOTAL](int2);

[T1_RSA_C_TOTAL T2_RSA_C_TOTAL T3_RSA_C_TOTAL](int3);

[T1_RSA_D_TOTAL T2_RSA_D_TOTAL T3_RSA_D_TOTAL](int4);

[T1_RSA_E_TOTAL T2_RSA_E_TOTAL T3_RSA_E_TOTAL](int5);

[T1_RSA_F_TOTAL T2_RSA_F_TOTAL T3_RSA_F_TOTAL](int6);

[t1* t2* t3*];

!here the parcels measurement errors are specified to be equal across the time!

T1_RSA_A_TOTAL T2_RSA_A_TOTAL T3_RSA_A_TOTAL (e1)

T1_RSA_B_TOTAL T2_RSA_B_TOTAL T3_RSA_B_TOTAL (e2)

T1_RSA_C_TOTAL T2_RSA_C_TOTAL T3_RSA_C_TOTAL (e3)

T1_RSA_D_TOTAL T2_RSA_D_TOTAL T3_RSA_D_TOTAL (e4)

T1_RSA_E_TOTAL T2_RSA_E_TOTAL T3_RSA_E_TOTAL (e5)

T1_RSA_F_TOTAL T2_RSA_F_TOTAL T3_RSA_F_TOTAl (e6);

! here we specified the parcel specific latent indicators, using parcel A as reference – that’s why it is not cite here; all the parcels’ indicators are fixed at one!

TB by T1_RSA_B_TOTAL@1 T2_RSA_B_TOTAL@1 T3_RSA_B_TOTAL@1;

TC by T1_RSA_C_TOTAL@1 T2_RSA_C_TOTAL@1 T3_RSA_C_TOTAL@1;

TD by T1_RSA_D_TOTAL@1 T2_RSA_D_TOTAL@1 T3_RSA_D_TOTAL@1;

TE by T1_RSA_E_TOTAL@1 T2_RSA_E_TOTAL@1 T3_RSA_E_TOTAL@1;

TF by T1_RSA_F_TOTAL@1 T2_RSA_F_TOTAL@1 T3_RSA_F_TOTAL@1;

TB TC TD TE TF WITH T1@0 T2@0 T3@0;

SAVEDATA: DIFFTEST IS strick.dat;

!Model for means!

Model:

T1 by

T1_RSA_A_TOTAL (a)

T1_RSA_B_TOTAL (b)

T1_RSA_C_TOTAL (c)

T1_RSA_D_TOTAL (d)

T1_RSA_E_TOTAL (e)

T1_RSA_F_TOTAL (f);

T2 by

T2_RSA_A_TOTAL (a)

T2_RSA_B_TOTAL (b)

T2_RSA_C_TOTAL (c)

T2_RSA_D_TOTAL (d)

T2_RSA_E_TOTAL (e)

T2_RSA_F_TOTAL (f);

T3 by

T3_RSA_A_TOTAL (a)

T3_RSA_B_TOTAL (b)

T3_RSA_C_TOTAL (c)

T3_RSA_D_TOTAL (d)

T3_RSA_E_TOTAL (e)

T3_RSA_F_TOTAL (f);

[T1_RSA_A_TOTAL@0 T2_RSA_A_TOTAL@0 T3_RSA_A_TOTAL@0];

[T1_RSA_B_TOTAL T2_RSA_B_TOTAL T3_RSA_B_TOTAL](int2);

[T1_RSA_C_TOTAL T2_RSA_C_TOTAL T3_RSA_C_TOTAL](int3);

[T1_RSA_D_TOTAL T2_RSA_D_TOTAL T3_RSA_D_TOTAL](int4);

[T1_RSA_E_TOTAL T2_RSA_E_TOTAL T3_RSA_E_TOTAL](int5);

[T1_RSA_F_TOTAL T2_RSA_F_TOTAL T3_RSA_F_TOTAL](int6);

!here means of the state latent factors are specified to be equal across the time!

[t1* t2* t3*] (means);

T1_RSA_A_TOTAL T2_RSA_A_TOTAL T3_RSA_A_TOTAL (e1)

T1_RSA_B_TOTAL T2_RSA_B_TOTAL T3_RSA_B_TOTAL (e2)

T1_RSA_C_TOTAL T2_RSA_C_TOTAL T3_RSA_C_TOTAL (e3)

T1_RSA_D_TOTAL T2_RSA_D_TOTAL T3_RSA_D_TOTAL (e4)

T1_RSA_E_TOTAL T2_RSA_E_TOTAL T3_RSA_E_TOTAL (e5)

T1_RSA_F_TOTAL T2_RSA_F_TOTAL T3_RSA_F_TOTAl (e6);

TB by T1_RSA_B_TOTAL@1 T2_RSA_B_TOTAL@1 T3_RSA_B_TOTAL@1;

TC by T1_RSA_C_TOTAL@1 T2_RSA_C_TOTAL@1 T3_RSA_C_TOTAL@1;

TD by T1_RSA_D_TOTAL@1 T2_RSA_D_TOTAL@1 T3_RSA_D_TOTAL@1;

TE by T1_RSA_E_TOTAL@1 T2_RSA_E_TOTAL@1 T3_RSA_E_TOTAL@1;

TF by T1_RSA_F_TOTAL@1 T2_RSA_F_TOTAL@1 T3_RSA_F_TOTAL@1;

TB TC TD TE TF WITH T1@0 T2@0 T3@0;

!model for means and variances of continuous latent factors!

Model:

T1 by

T1_RSA_A_TOTAL (a)

T1_RSA_B_TOTAL (b)

T1_RSA_C_TOTAL (c)

T1_RSA_D_TOTAL (d)

T1_RSA_E_TOTAL (e)

T1_RSA_F_TOTAL (f);

T2 by

T2_RSA_A_TOTAL (a)

T2_RSA_B_TOTAL (b)

T2_RSA_C_TOTAL (c)

T2_RSA_D_TOTAL (d)

T2_RSA_E_TOTAL (e)

T2_RSA_F_TOTAL (f);

T3 by

T3_RSA_A_TOTAL (a)

T3_RSA_B_TOTAL (b)

T3_RSA_C_TOTAL (c)

T3_RSA_D_TOTAL (d)

T3_RSA_E_TOTAL (e)

T3_RSA_F_TOTAL (f);

[T1_RSA_A_TOTAL@0 T2_RSA_A_TOTAL@0 T3_RSA_A_TOTAL@0];

[T1_RSA_B_TOTAL T2_RSA_B_TOTAL T3_RSA_B_TOTAL](int2);

[T1_RSA_C_TOTAL T2_RSA_C_TOTAL T3_RSA_C_TOTAL](int3);

[T1_RSA_D_TOTAL T2_RSA_D_TOTAL T3_RSA_D_TOTAL](int4);

[T1_RSA_E_TOTAL T2_RSA_E_TOTAL T3_RSA_E_TOTAL](int5);

[T1_RSA_F_TOTAL T2_RSA_F_TOTAL T3_RSA_F_TOTAL](int6);

!here the variances of the state latent factor are specified to be equal across the three time points!

t1 t2 t3 (Var_tau);

!here the means of the state latent factor are specified to be equal across the three time points!

[t1* t2* t3*](means);

T1_RSA_A_TOTAL T2_RSA_A_TOTAL T3_RSA_A_TOTAL (e1)

T1_RSA_B_TOTAL T2_RSA_B_TOTAL T3_RSA_B_TOTAL (e2)

T1_RSA_C_TOTAL T2_RSA_C_TOTAL T3_RSA_C_TOTAL (e3)

T1_RSA_D_TOTAL T2_RSA_D_TOTAL T3_RSA_D_TOTAL (e4)

T1_RSA_E_TOTAL T2_RSA_E_TOTAL T3_RSA_E_TOTAL (e5)

T1_RSA_F_TOTAL T2_RSA_F_TOTAL T3_RSA_F_TOTAl (e6);

!A by T1_RSA_A_TOTAL T2_RSA_A_TOTAL T3_RSA_A_TOTAL;

TB by T1_RSA_B_TOTAL@1 T2_RSA_B_TOTAL@1 T3_RSA_B_TOTAL@1;

TC by T1_RSA_C_TOTAL@1 T2_RSA_C_TOTAL@1 T3_RSA_C_TOTAL@1;

TD by T1_RSA_D_TOTAL@1 T2_RSA_D_TOTAL@1 T3_RSA_D_TOTAL@1;

TE by T1_RSA_E_TOTAL@1 T2_RSA_E_TOTAL@1 T3_RSA_E_TOTAL@1;

TF by T1_RSA_F_TOTAL@1 T2_RSA_F_TOTAL@1 T3_RSA_F_TOTAL@1;

TB TC TD TE TF WITH T1@0 T2@0 T3@0;

!model for means, variances, and covariances!

Model:

T1 by

T1_RSA_A_TOTAL (a)

T1_RSA_B_TOTAL (b)

T1_RSA_C_TOTAL (c)

T1_RSA_D_TOTAL (d)

T1_RSA_E_TOTAL (e)

T1_RSA_F_TOTAL (f);

T2 by

T2_RSA_A_TOTAL (a)

T2_RSA_B_TOTAL (b)

T2_RSA_C_TOTAL (c)

T2_RSA_D_TOTAL (d)

T2_RSA_E_TOTAL (e)

T2_RSA_F_TOTAL (f);

T3 by

T3_RSA_A_TOTAL (a)

T3_RSA_B_TOTAL (b)

T3_RSA_C_TOTAL (c)

T3_RSA_D_TOTAL (d)

T3_RSA_E_TOTAL (e)

T3_RSA_F_TOTAL (f);

[T1_RSA_A_TOTAL@0 T2_RSA_A_TOTAL@0 T3_RSA_A_TOTAL@0];

[T1_RSA_B_TOTAL T2_RSA_B_TOTAL T3_RSA_B_TOTAL](int2);

[T1_RSA_C_TOTAL T2_RSA_C_TOTAL T3_RSA_C_TOTAL](int3);

[T1_RSA_D_TOTAL T2_RSA_D_TOTAL T3_RSA_D_TOTAL](int4);

[T1_RSA_E_TOTAL T2_RSA_E_TOTAL T3_RSA_E_TOTAL](int5);

[T1_RSA_F_TOTAL T2_RSA_F_TOTAL T3_RSA_F_TOTAL](int6);

!here the variances of the state latent factor are specified to be equal across the three time points!

t1 t2 t3 (Var_tau);

!here the means of the state latent factor are specified to be equal across the three time points!

[t1* t2* t3*](means);

!here the covariances of the state latent factor are specified to be equal across the three time points!

t1-t3 with t1-t3 (cov_tau);

T1_RSA_A_TOTAL T2_RSA_A_TOTAL T3_RSA_A_TOTAL (e1)

T1_RSA_B_TOTAL T2_RSA_B_TOTAL T3_RSA_B_TOTAL (e2)

T1_RSA_C_TOTAL T2_RSA_C_TOTAL T3_RSA_C_TOTAL (e3)

T1_RSA_D_TOTAL T2_RSA_D_TOTAL T3_RSA_D_TOTAL (e4)

T1_RSA_E_TOTAL T2_RSA_E_TOTAL T3_RSA_E_TOTAL (e5)

T1_RSA_F_TOTAL T2_RSA_F_TOTAL T3_RSA_F_TOTAl (e6);

TB by T1_RSA_B_TOTAL@1 T2_RSA_B_TOTAL@1 T3_RSA_B_TOTAL@1;

TC by T1_RSA_C_TOTAL@1 T2_RSA_C_TOTAL@1 T3_RSA_C_TOTAL@1;

TD by T1_RSA_D_TOTAL@1 T2_RSA_D_TOTAL@1 T3_RSA_D_TOTAL@1;

TE by T1_RSA_E_TOTAL@1 T2_RSA_E_TOTAL@1 T3_RSA_E_TOTAL@1;

TF by T1_RSA_F_TOTAL@1 T2_RSA_F_TOTAL@1 T3_RSA_F_TOTAL@1;

TB TC TD TE TF WITH T1@0 T2@0 T3@0;
